# 

**Published:** 1866-04

**Authors:** 


### CONTENTS.

ORIGINAL COMMUNICATIONS.

Dental Materia Medica.... 147
Valedictory Address.... 153
Sixth Year Molars.... 161
Address by Geo. H. Cushing, D. D. S.... 164
Address by Dr. J. Chesebrough.... 171
Dr. Bogues improvement upon the Kingsley Artificial Palate.... 176

PROCEEDINGS OF SOCIETIES.

Synopsis of Discussions, Missouri State Dental Society.... 180

SELECTIONS.

Thoroughness in Dental Operations.... 186
Death from Chloroform.... 188
Chicago Dental Society.... 189
Northern Ohio Dental Association.... 189
Western New York Dental Association.... 190

EDITORIAL.

A slight Eulogistic Mistake.... 190
Professional.... 191
Personal.... 192
Marriage.... 192
Died.... 192

